# The quality of primary care provided to the elderly in Israel

**DOI:** 10.1186/s13584-018-0214-3

**Published:** 2018-06-04

**Authors:** Rachel Podell, Vered Kaufman Shriqui, Yael Wolff Sagy, Orly Manor, Arie Ben-Yehuda

**Affiliations:** 1 Program team of the National Program for Quality Indicators in Community Healthcare in Israel, Hebrew University, POB 12272, 92210 Jerusalem, Israel; 2 Braun School of Public Health, Hebrew University, POB 12272, 92210 Jerusalem, Israel; 3 Department of Nutritional Sciences, School of Health Sciences, Ariel University, Ariel, Israel; 4 Program directorate of the National Program for Quality Indicators in Community Healthcare in Israel, Hebrew University, POB 12272, 92210 Jerusalem, Israel; 5 Braun School of Public Health, Hebrew University, POB 12272, 92210 Jerusalem, Israel; 6 Program directorate of the National Program for Quality Indicators in Community Healthcare in Israel, Hadassah Medical Center, POB 12000, 92210 Jerusalem, Israel

**Keywords:** Quality indicators, Community healthcare, Elderly health, Gender disparities, Vaccinations, Underweight, Benzodiazepine overuse

## Abstract

**Background:**

In view of increasing global and local trends in population ageing and the high healthcare utilization rates among the elderly, this study assesses the quality of primary care provided to the elderly population in Israel. It examines changes in quality over time, how quality varies across sub-groups of the elderly, and how quality in Israel compares with other countries. Data originate from the National Program for Quality Indicators in Community Healthcare (QICH), which operates in full collaboration with Israel’s four HMOs.

**Methods:**

The study population included all elderly Israeli residents aged 65 years or older during 2002–2015 (*N* = 879,671 residents in 2015). Seven elderly-specific quality indicators from within the QICH framework were included: influenza and pneumococcal vaccinations, benzodiazepine overuse, long-acting benzodiazepine use, body weight documentation, weight loss and underweight. In addition, two non-age specific quality indicators relating to diabetes mellitus were included: the rate of HbA1C documentation and uncontrolled diabetes. Data were collected from patient electronic medical records (EMR) in accordance with each HMO, and aggregated by three variables: gender, age, and socio-economic position (SEP).

**Results:**

During the measurement period, vaccination rates significantly increased (Influenza: from 42.0% in 2002 to 63.2% in 2015; and pneumococcal vaccination: from 25.8% in 2005 to 77.0% in 2015). Body weight documentation (in 65–74 year old persons) increased from only 16.3% in 2003 to 80.9% in 2015. The rate of underweight (BMI < 23 kg/m^2^) and significant weight-loss (10% or more of their body weight) was only measured in 2015. The overall rate of benzodiazepine overuse remained steady from 2011 to 2015 at around 5%, while the rate of long-acting benzodiazepine use decreased from 3.8% in 2011 to 2.4% in 2015.

The rate of HbA1c documentation for elderly diabetics was higher than for non-elderly diabetics in 2015 (92.2% vs 87.9%). The rate of uncontrolled diabetes was lower for the elderly than the non-elderly population in 2015 (6.9% vs. 15.7%).

Gender disparities were observed across all measures, after age stratification, with worse indicator rates among females compared to males. SEP-disparities were not consistent across measures.

In all indicators except benzodiazepine overuse, Israel showed a higher quality of care for the elderly in comparison with the international healthcare community.

**Conclusions:**

Overall, the quality of care received by elderly Israelis has improved substantially since measurements first began; yet, females receive lower quality care than males. Monitoring results of primary care quality indicators can contribute to population’s successful aging; both chronic conditions at earlier ages (e.g. diabetes), and short-term hazardous conditions such as the use of potentially harmful medications and weight loss should be evaluated.

## Background

Worldwide population ageing, due to an increase in life expectancy and decrease in fertility rates, is occurring rapidly [1]. The World Health Organization (WHO) projects by 2050 the world’s older population (60 years or above, WHO definition) will encompass 22% of the total population [2]. Correspondingly, 17% of the overall European population in 2014 was comprised of adults 65 years or older with growth expected in the future [3]. By the year 2030, more than 20% of the US population is expected to be 65 years and older [4].

While modern health improvements help reduce serious disabilities in the elderly, WHO reports an increase in multiple morbidities, burden of chronic diseases, and health-care utilization [1, 5]. Chronic disease affects 61% of Europeans 65 years or older [6], and multiple morbidities affect more than 60% of elderly adults worldwide [7]. Two out of three older Americans suffered from multiple chronic conditions in 2013 [8]. Health care utilization in high-income countries increases with age; in Canada, the elderly accounted for 40% of acute hospital stays in 2010 [9]. In addition, healthcare expenditure peaked for adults 65–74 years in the United Kingdom, but decreased until the last year of life. This last year is the strongest driver of increasing healthcare expenditures worldwide [1, 10].

Similar to the global ageing rate, Israel is experiencing a rapid rate of elderly population growth. The proportion of the elderly among the general population is expected to increase to 15% by 2035 (11% in 2014). In 2014, one in four households in Israel included someone 65 years or older [3]. Israeli immigration trends have also contributed to both the relative and absolute increase in the number of older adults [11].

Chronic disease affected approximately 70% of elderly Israelis in 2009 [12]. A study conducted by the second largest health maintenance organization in Israel (Maccabi Health Services), found that over 90% of study participants 75 years or older suffered from multiple chronic conditions [13]. Following global healthcare utilization trends, the elderly population (65 years or older) was hospitalized 3.2 times more than the general population (2013) [3] and made on average 11.2 primary care physician visits per year (versus an overall average of only three per year for the general population) [11].

Aging affects both mental and physical health, promoting the study of the quality of care among the elderly in Israel. Past elderly studies have focused on primary care (e.g. the chronic care model or home-based primary care) [14, 15], elderly disease states [16], or elderly care processes within the health system [17]. This research aims to add new knowledge to the field. This study sought to examine the quality of primary care provided to elderly Israelis over a 14-year measurement period utilizing quality indicators from within the framework of the Israel National Program for Quality Indicators in Community Healthcare (QICH) [18].

## Methods

The Israeli National Health Insurance (NHI) law was implemented in 1995 offering all Israeli residents a standardized basket of medical services through four health maintenance organizations (HMOs*, kupot cholim*). Under this law, every resident has the right to choose their HMO, transfer from one HMO to another, and receive health services through their HMO [19]. QICH works with the four HMOs and evaluates the quality of community-based medical care in Israel, provides this information to policy makers and the public, promotes health care monitoring and guideline-based care, and improves population health. QICH monitors all Israeli citizens, including the entire Israeli elderly population. This study selected seven elderly-specific (65 years or older) quality indicators available from within the QICH framework (Table 1).

**Table 1:** QICH elderly-specific quality indicators

Quality indicator	Denominator	Numerator	Comments
Influenza Vaccination	All residents aged 65 years and older during the measurement year	Number of residents (out of the denominator) who received a seasonal influenza vaccination during the measurement year	• “Seasonal” refers to the winter season during the height of the influenza virus, September 1st of the former year to February 28th of current year • Information derived from pharmacy claims and nursing notes
Pneumococcal Vaccination	All residents aged 65–74 years during the measurement year	Number of residents (out of the denominator) who received the pneumococcal vaccination either once after age 65 or in the last 5 years	• Information derived from pharmacy claims and nursing notes • The 65–74 year age group was selected due to data availability being limited to the past 10 years
Body Weight and Height Documentation	All residents aged 65–84 years during the measurement year	Number of residents (out of the denominator) with documented body weight during the measurement year and at least one height documentation between 65 and 84 years of age	• Information derived from medical appointment documentation
Underweight	Number of residents aged 65 or older during the measurement year with body weight and height documentation (see above)	Number of residents (out of the denominator) with most recent documented BMI < 23 kg/m^2^	• Information derived from medical appointment documentation
Weight loss	All residents aged 65 years or older during the measurement year	Number of residents (out of the denominator) experiencing a decline of 10% or more in their body weight within 2 years	• Information derived from medical appointment documentation
Benzodiazepine Overuse	All residents aged 65 years and older during the measurement year	Number of residents (out of the denominator) with benzodiazepine overuse during the measurement year	• Overused benzodiazepines defined by purchased benzodiazepines or related drugs ≥ 365 DDD/year in the measurement year (DDD = Defined Daily Dose) • Information derived from pharmacy claims
Benzodiazepine Long-Acting Use	All residents aged 65 years and older during the measurement year	Number of residents (out of the denominator) with at least one purchase of long-acting benzodiazepines during the measurement year	• Long-acting benzodiazepine use defined by the purchase of one or more long-acting benzodiazepines or related drugs • Information derived from pharmacy claims

### Population

The study population included all elderly residents aged 65 years or older during the years 2002–2015. The study population grew from 591,877 residents in 2002 to 879,671 residents in 2015.

### Data

Data for 2002–2015 were collected from patient electronic medical records (EMR) in accordance with the four HMOs. Yearly data for residents who passed away, elderly residents who switched HMOs (0.28%, 2014), and missing EMR data (0.7%, 2015) were not included in the dataset. The source of relevant study information included physician visits, nursing notes, pharmacy claims, medical appointments, and immunization records. To ensure confidentiality, data from each HMO were anonymized, aggregated, and merged into a national dataset to calculate population-wide rates.

Data were aggregated by three variables: gender, age groups, and socio-economic position (SEP). SEP was defined by co-payment exemption when receiving health services, classified as either low SEP (exempt group, representing 39% of the elderly population in 2015) or middle and high SEP (non-exempt group). In the documentation period, exemption was granted based on national insurance allowances such as income support, handicap allowance, large family allowance, etc. In a pilot study, the above SEP indicator was validated against an area-based SEP variable. Using census information on numerous indicators (including income, education and unemployment) the Israeli Central Bureau of Statistics routinely calculate and allocate a socioeconomic score to each geographical statistical area (GSA). In the pilot study, subjects received the score allocated to their GSA (based on their address as recorded in the HMO) [20]. Validation showed a strong association between these two variables; 52% of lower quartile GSA-based SEP had an exemption from co-payments and 24% of those in the higher quartile were exempted.

### Quality indicators

The QICH indicators undergo a three-step evaluation system before implementation. The program’s directorate consults evidence, guidelines, international measures, and professional recommendations. Then, an internal HMO assessment is conducted, followed by hearings with stakeholders (e.g. Health Councils, Health Associations, and clinical experts) directed by the steering committee. Five main selection criteria are utilized to evaluate the indicators: (1) importance and relevance to the field, (2) sufficient evidence to support the indicator, (3) quantifiability, (4) availability and accessibility of electronic data from the EMRs, and (5) ability to implement within the healthcare setting. Indicators meeting these criteria undergo a consensus decision for incorporation into the QICH framework and then definitions are unified across HMOs. The QICH quality indicator development process is similar to the recommendations provided by the US Institute of Medicine [21]. Seven elderly-specific (65 years and older) indicators, which underwent this incorporation process into the QICH framework, were selected for this study. Detailed definitions of the indicators are given in Table 1; influenza and pneumococcal vaccinations, benzodiazepine overuse, long-acting benzodiazepine use, body weight documentation, weight loss and underweight. The quality indicators included indicated either measurements (i.e. weight, height and level of HbA1C) from which quality indicators were later calculated, purchasing of benzodiazepines, or procedures (i.e. vaccinations). Despite the fact that the information on body weight existed in the EMRs of HMOs from 2003, the indicators of underweight and weight-loss were only calculated from 2013, the year the quality indicators steering committee has decided to incorporate them in the quality indicators program. The comprehensive geriatric assessment indicator was not included in QICH due to lack of evidence supporting some components of this assessment and the variability of use among clinicians.

*Influenza and pneumococcal vaccinations* aim to prevent influenza and pneumococcal infections in the elderly; these diseases are major causes of morbidity and mortality in this population. In an influenza season, approximately 90% of influenza-related deaths occur in the elderly [22, 23]. During the 2014–2015 Israeli influenza season, the rate of clinic visits for those 65 years and older with an influenza-like illness (ILI) was higher than the previous two influenza seasons, despite a higher rate of influenza vaccinations [24]. The incidence rate of invasive pneumococcal disease (IPD) in Israel between 2009 and 2010 for those aged 65–74 years was 20 per 100,000 people; incidence rate increased as age increased. The case-fatality rate was 25% among 65–74 years-old, and 35% among those 85 years-old and older [11]. Additionally, half of all IPD cases occurred in those 65 years or older in the United States in 2013 [25]. This study sought to include the pneumococcal vaccinations indicator, despite weak supporting evidence, due to its public health importance and inclusion in elderly primary care guidelines of most countries. Inclusion of these indicators was supported by the Israeli Ministry of Health [26] and the US Centers for Disease Control and Prevention [27] recommendation for a seasonal influenza vaccination once a year for adults 65 years or older, and a pneumococcal vaccination once after age 65. Further, herd immunity through vaccinated children is less effective than directly vaccinating elderly adults [28].

*Body weight documentation and detection of underweight and weight loss in the elderly* are important steps to preventing morbidity and mortality. Documenting body weight is essential to preventative care, as is providing information on patients’ weight status compared to the recommended healthy weight status, nutritional needs, medication dosing, and implied specific health problems [29]. Underweight in the elderly has been associated with excess mortality, versus those with a normal weight [30]. Instability in elderly weight has also been associated with all-cause mortality [31], and monitoring elderly weight changes over time assists in understanding elderly health quality. Moreover, dynamic body weight measures (i.e. weight changes), compared to static body weight measures (e.g. body mass index), better predicts mortality among the elderly [31]. Weight loss has been associated with elderly health conditions, such as frailty. Frailty is associated with increased odds for falls, hospitalizations, longer hospital stays, delayed surgery recovery, disability, and death [32, 33]. According to the US HMO Medicare coverage and eligibility guidelines, elderly patients should receive a body weight measurement at every yearly “wellness” visit [34]. Weight loss was defined under QICH framework as the calculated difference between two consecutive measurements, whereas for the definition of underweight we used the cutoff value of Body Mass Index (BMI, which is person’s weight in kilograms divided by the square of height in meters) lower than 23.

*Overuse of benzodiazepines and any use of long-acting benzodiazepines* are important elderly population measures. Benzodiazepines are a veteran class of medications with a primary usage for sleep and anti-anxiety, also causing confusion, memory loss, loss of focus and balance leading to falls, dependency and withdrawal symptoms, motor vehicle accidents, and hip fractures in the elderly population [35–37]. The slower metabolic rate of the elderly population causes benzodiazepines to build-up in the bloodstream leading to a prolonged exertion of the medication effect [37], and resulting in elderly specific morbidity. Benzodiazepine prescribing is contra-indicated among the elderly for insomnia, agitation, or delirium and this indicator was created in accordance with the OECD measures [38–40]. The benzodiazepine indicators are relevant today since studies have found that benzodiazepine prescribing and usage in the elderly remains high, despite guideline recommendations for decrease or stoppage of use. A review performed on the usage of benzodiazepines in the United Kingdom, United States, and Europe, found that as age increased overall benzodiazepine consumption increased, with higher usage rates in women compared to men [35]. A recent US study found that 8.7% of the study participants aged 65–80 years used benzodiazepines and 23.8% of that cohort had long-acting benzodiazepine use [41]. A 2007 Australian study reported 15.7% of elderly Australian study participants had at least one benzodiazepine prescription [36].

Additionally, we examined the performance on two key quality indicators in the field of diabetes treatment in 2015, which are not age-specific in order to establish whether performance on these measures differs between the elderly and the non-elderly. For these measures, we also examined the change in performance rate during a five-year period, 2011–2015: documentation rate of hemoglobin A1c (HbA1c) levels for individuals with diabetes mellitus, and the rate of uncontrolled diabetes mellitus (defined as HbA1c greater than 9%).

### Analysis

Annual trends were examined for each indicator for the entire measurement period. Data for 2015 were stratified by gender, age, and socio-economic position (SEP), and differences in each indicator’s performance rate by these socio-demographic variables were assessed using a z-typed test. To adjust for multiple comparisons (20 tests were conducted) and the large sample, a *p*-value below 0.0005 was considered statistically significant.

### Quality of the data

The definitions of the included indicators remained unchanged during the measurement period, as well as the data extraction methods, allowing a reliable examination of time-trends. Additionally, the methods created for data collection includes an extensive evaluation program intended to minimize the chance of various errors, including differences between health plans in documentation and coding of their insured population’s characteristics. Data extraction undergoes a three-level audit, including: 1) extensive internal quality assurance tests conducted by the HMOs, 2) QICH audit and cross-examination of the reported data, and 3) a thorough external audit. This method has certainly led to fewer errors, although unable to eliminate them entirely.

### International comparisons

Results were also compared to similar data from Organization for Economic Cooperation and Development (OECD) and other developed countries. Definitions of the rate of Pneumococcal and Influenza immunization were comparable across countries. The definitions of the rate of benzodiazepine overuse, and long-term benzodiazepine use were similar, yet whereas in most OECD countries prescriptions of benzodiazepines were counted, the Israeli data allowed a more accurate evaluation using pharmacy claims of benzodiazepines [18]. The rate of body weight documentation was not reported in the countries considered and the definition of the rate of underweight or weight-loss in the elderly, varied between QICH and other developed countries. While across OECD countries the prevalence of underweight, was defined as BMI < 18.5 kg/m^2^, QICH directorate, following consultation with Israeli experts, choose to take a public health perspective and identify individuals at risk of underweight and thus used BMI ≤ 23 kg/m^2^ [42].

## Results

**Fig. 1:**
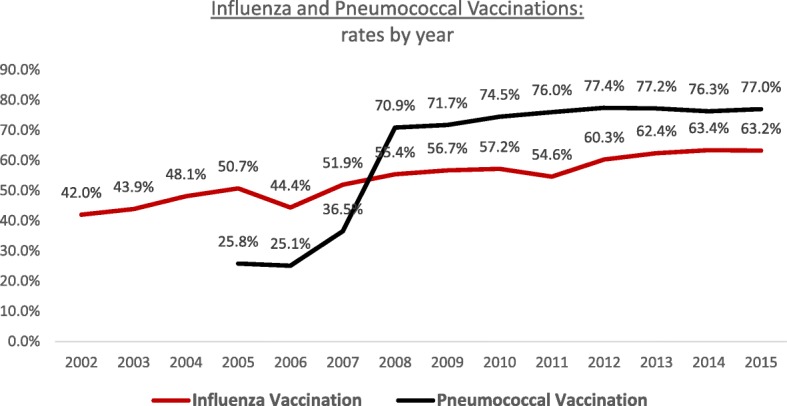
Influenza and pneumococcal vaccinations among Israelis aged 65 years or older, rates by year, 2002–2015. Pneumococcal vaccination was defined as those who received the vaccination once in the last 6 years through 2007, since 2008 it is defined by the current definition

Since the first measurement in 2002, overall influenza vaccination rates increased from 42.0% to 63.2% in 2015 (Fig. 1). In 2015, vaccination rate was lowest for those in the 65–74 years age group (59.5%) and highest for those aged 75–84 years (68.0%), *p* < 0.00001 (Table 2). Females were vaccinated less than males (61.4% vs 65.6%, *p* < 0.00001) in all age groups (females aged 65–74 years were the least vaccinated (58.0%), Appendix). The absolute difference of immunization rate between males and females increased with age (from 3.2% for those aged 65–74 years to 7.9% for those 85 years or older, data not shown). SEP groups showed similar vaccination rates; however, the males of low SEP were vaccinated the most, while females of low SEP were vaccinated the least (67.6% vs. 60.6%, Appendix).

**Table 2:** Quality indicator performance rates by gender, socioeconomic position, and age, 2015

		Denominator	Numerator	Rate
Influenza vaccination				
Total		879,671	556,355	63.25%
Gender*	Male	385,492	252,918	65.61%
	Female	494,179	303,437	61.40%
SEP	Low	340,395	215,794	63.40%
	Middle & High	539,276	340,561	63.15%
Age*	65–74	488,997	290,925	59.49%
	75–84	283,209	192,595	68.00%
	85 or above	107,465	72,835	67.78%
Pneumococcal vaccination				
Total		449,274	346,032	77.02%
Gender*	Male	208,638	164,901	79.04%
	Female	240,636	181,131	75.27%
SEP*	Low	136,138	110,501	81.17%
	Middle & High	313,136	235,531	75.22%
Age	65–74	449,274	346,032	77.02%
	75–84	NA	NA	NA
	85 or above	NA	NA	NA
Benzodiazepine overuse				
Total		879,671	45,858	5.21%
Gender*	Male	385,492	15,402	4.00%
	Female	494,179	30,456	6.16%
SEP*	Low	340,395	23,841	7.00%
	Middle & High	539,276	22,017	4.08%
Age*	65–74	488,997	14,567	2.98%
	75–84	283,209	18,946	6.69%
	85 or above	107,465	12,345	11.49%
Long-term benzodiazepine				
Total		879,671	21,469	2.44%
Gender*	Male	385,492	7801	2.02%
	Female	494,179	13,668	2.77%
SEP*	Low	340,395	9039	2.66%
	Middle & High	539,276	12,430	2.30%
Age*	65–74	488,997	10,949	2.24%
	75–84	283,209	7668	2.71%
	85 or above	107,465	2852	2.65%
BMI documentation				
Total		770,565	623,731	80.94%
Gender*	Male	345,894	281,149	81.28%
	Female	424,671	342,582	80.67%
SEP*	Low	266,803	221,828	83.14%
	Middle & High	503,762	401,903	79.78%
Age*	65–74	485,473	386,987	79.71%
	75–84	284,963	236,744	83.08%
	85 or above	NA	NA	NA
Underweight				
Total		703,827	90,632	12.88%
Gender*	Male	312,365	36,537	11.70%
	Female	391,462	54,095	13.82%
SEP*	Low	257,456	31,455	12.22%
	Middle & High	446,371	59,177	13.26%
Age*	65–74	386,936	43,546	11.25%
	75–84	237,614	30,753	12.94%
	85 or above	79,277	16,333	20.60%
Weight loss				
Total		613,408	37,673	6.14%
Gender*	Male	272,010	14,968	5.50%
	Female	341,398	22,705	6.65%
SEP*	Low	222,945	15,613	7.00%
	Middle & High	390,463	22,060	5.65%
Age*	65–74	320,649	16,165	5.04%
	75–84	221,014	14,915	6.75%
	85 or above	71,745	6593	9.19%

All rates are calculated as crude rates

*p < 0.00001

Overall rates of pneumococcal vaccination increased since the first measurement in 2005 (25.8% to 77.0% in 2015, Fig. 1). Vaccination rates increased dramatically from 36.5% in 2007 to 70.9% in 2008 due to excessive immunization efforts of the HMOs. In 2015, females were vaccinated at a lower rate than males (75.3% vs 79.0%, *p* < 0.00001); individuals of low SEP were vaccinated more compared to the individuals of the middle and high SEP (81.2% vs 77.0%, *p* < 0.00001) (Table 2). Males of low SEP were vaccinated at the highest rate, while females of high and middle SEP were vaccinated at the lowest rate (84.0% vs. 75.3%, Appendix).

**Fig. 2:**
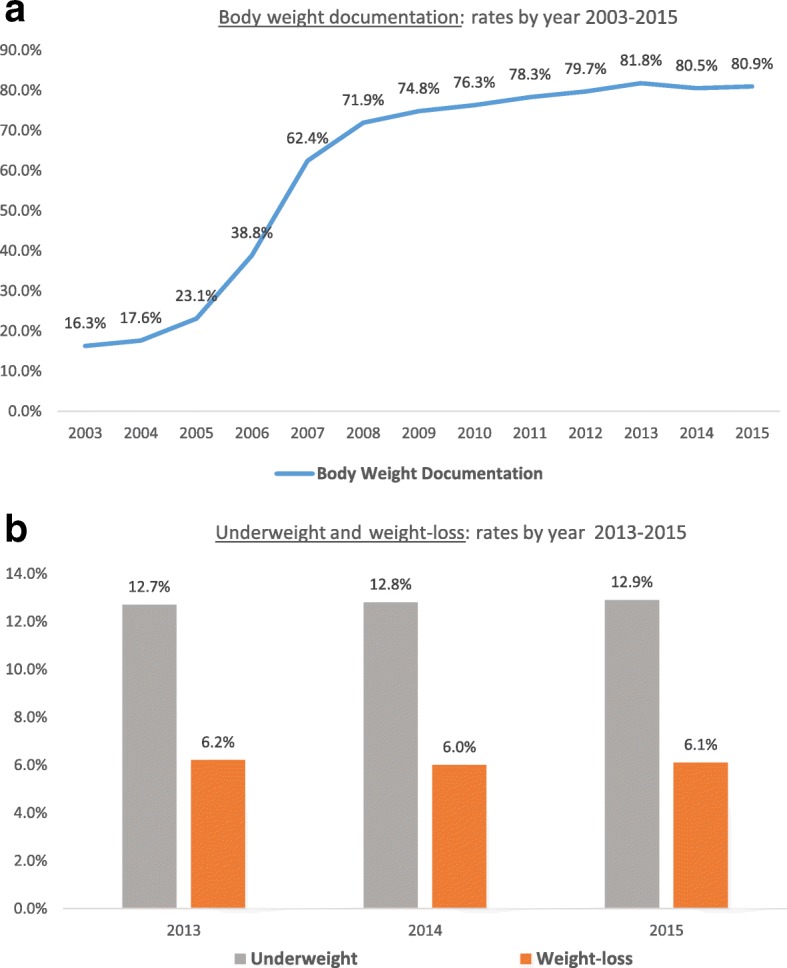
**a** Body weight documentation, rates by year, 2003-2015; **b** Underweight and weight-loss, rates by year, 2013-2015; All rates are calculated as crude rates; Body weight was measured for elderly 65-74 years until 2010; in 2011 body weight was measured for those 65 to 84 years-old

Body weight documentation was first measured in 2003 (16.3%, only for the 65–74 age group). By 2015, the rate reached 80.9% in those aged 65–84 years (Fig. 2). In 2015, males and females had similar rates of documentation (Table 2). Higher documentation rates were seen among those with low SEP (83.1%) compared to the middle and high SEP population (79.8%, *p* < 0.00001, Table 2). Documentation rate was lower for those aged 65–74 (79.7%) compared to the older age group (83.1% among those 75–84 years-old, *p* < 0.00001, Table 2).

Underweight and weight-loss were calculated for the first time during the period 2013–2015 and showed stability over this period.

The rate of underweight (BMI < 23 kg/m^2^) among the elderly Israeli population in 2015 was 12.9%. The prevalence of underweight was higher in female compared to male (13.8% vs 11.7%, respectively, *p* < 0.00001, Table 2). This gender-difference was mostly pronounced within the younger age group (65–74 years-old), with an absolute difference of nearly 3% in the prevalence of underweight between female and male; while less than 1% difference between genders was found in the older age groups (0.4% in 75–84 years-olds, and 0.8% in 85 years-old or older, data not shown). Individuals of low SEP were less likely to be classified as underweight, compared to those of middle-high SEP (12.2% vs 13.3%, respectively, *p* < 0.00001, Table 2). The prevalence of underweight significantly increased with increasing age (from 11.3% among those 65–74 years-old, and up to 20.6% among those aged 85 or older, *p <* 0.00001, Table 2), a trend observed regardless of gender and SEP (Appendix).

The rate of elderly persons who experienced a significant weight-loss (10% or more of their body weight) within 2 years, was 6.1% in 2015. The rate was higher among females compared to males (6.7% vs 5.5%, *p* < 0.00001, Table 2), with a consistent absolute difference between genders (of approximately 1%) across age and SEP groups (Appendix). Individuals of low SEP had higher rates of weight-loss compared to individuals of middle-high SEP (7.0% vs 5.6%, *p* < 0.00001, Table 2). Similar to underweight, the rates also increased with increasing age from 5.0% among those 65–74 years-old, and up to 9.2% among those aged 85 or older (Table 2).

**Fig. 3:**
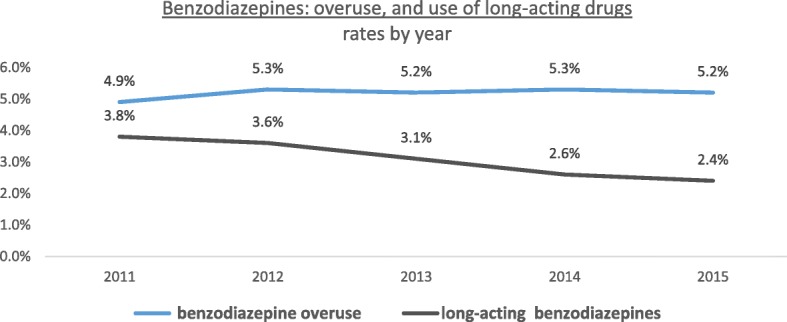
Benzodiazepine usage among Israelis aged 65 years or older, rates by year, 2011–2015

Overall rate of benzodiazepine overuse remained steady from 2011, the first year of measurement, to 2015 at around 5% (Fig. 3). In 2015, the 85 years or older population overused benzodiazepines at the highest rate (11.5%, compared to only 3.0% among those 65–74 years old, *p* < 0.00001, Table 2) and females overused benzodiazepines at a higher rate than males (6.2% vs 4.0%, respectively, *p* < 0.00001, Table 2). The low SEP population overused benzodiazepines at a much higher rate compared to the middle-high SEP population (7.0% compared to 4.1%, *p* < 0.00001, Table 2).

The rate of long-acting benzodiazepine use decreased since first measured in 2011, from 3.8% to 2.4% in 2015 (Fig. 2). In 2015, females used long-acting benzodiazepine at a higher rate compared to males (2.8% vs 2.0%, *p* < 0.00001, Table 2). Use was slightly more frequent among individuals of low SEP vs. those of middle-high SEP (2.7% vs. 2.3%, *p* < 0.00001), and higher among individuals aged 75 or older compared to the youngest age group (2.7% vs 2.2%, *p* < 0.00001, Table 2).

Additionally, we examined two quality indicators, which are not age specific in order to establish whether these measures differ between the elderly and the non-elderly. In 2015, *N* = 234,349 individuals aged 18–64 years, and *N* = 253,823 individuals aged 65 years or above were diabetics.

### Documentation rate of hemoglobin A1c (HbA1c) levels for individuals with diabetes mellitus

In 2015, the rate of HbA1c documentation (at least once during the measurement year), was higher among the elders (92.2%) compared to the non-elders (87.9%). In addition, when looking back at the five previous years (2011 to 2015), the rates were steady among the elders (91.6% in 2011) and among non-elders alike (88.0% in 2011).

### Rate of uncontrolled diabetes mellitus (HbA1c greater than 9%)

In 2015, the rate of uncontrolled diabetes (defined as HbA1c > 9% in the last measurement during the year) was 2.3 times lower among the elders (6.9%) compared to non-elders (15.7%). This measure has significantly improved since measurements began in 2002 (data not shown). Between 2011 and 2015, the rates of uncontrolled diabetes dropped by 14.5% (relative change, from 18.4% in 2011 to 15.7% in 2016) among those aged 18–64, and by 20.7% among those 65 years and above (from 8.7% in 2011 to 6.9% in 2015), showing a remarkably higher rate of improvement among elders.

## Discussion

Overall, elderly quality of care improved since measurements first began. Yet, quality of healthcare is lacking among certain socio-demographic groups within the framework of the Israeli elderly population. Influenza vaccination rates increased in the last 12 years, yet elderly women experienced the lowest quality of care. Similarly, overall rates of pneumococcal vaccinations increased substantially in the last 8 years; however, females again received lower quality of care. Interestingly, individuals of low SEP received pneumococcal vaccinations at a higher rate compared to the middle and high SEP population. The rate of body weight documentation increased in an 11-year measurement period. It should be noted that during this period, improvement in EMR took place, hence facilitating digital documentation, and contributing to the increase in height and weight documentation.

In contrast to the other indicators, females and males had very similar rates of documentation. Underweight and significant weight-loss were more frequent among females than among males, and substantially increased with increasing age.

Rates of benzodiazepine overuse remained steady in a three-year measurement period, while rates of long-acting benzodiazepine use decreased. Females and those 85 years or older overused benzodiazepines at the highest rate and females had the highest rate of long-acting benzodiazepine use.

Two key quality indicators in the field of diabetes care showed better performance among the elderly compared to the non-elderly population in 2015. Also, when comparing changes by age group along a five-year timeline, changes in rates for the elderly group were either similar to or better than the non-elderly group (stability in HbA1c test performance, steeper decrease in the rate of uncontrolled diabetes). Altogether, the diabetes control indicators show that the quality of care delivered to the elderly in Israel is comparable to that given to the younger population or even better.

The Israeli elderly population has some distinct characteristics compared to the international elderly population. Israel’s high life expectancy is a main contributor to the growing elderly population. In 2014, life expectancy at birth in Israel was 80.2 years for males, ranking in the top five countries with the highest life expectancy, and 84.1 for females [43]. In addition, Israel has a very low rate of institutionalization among the elderly population, only 3% of adults aged 65 or older live in long-term care institutions [3]. Correspondingly, the 97% of the population who live within the community experience a high rate of social support, allowing the elderly the ability to succeed in this environment. Furthermore, the composition of the elderly Israeli population sees diversity through immigrants (only 28% of the older population were born in Israel), Jews, and Arabs (8% of population, 2014) [11]. Lastly, while the rate of elderly growth is increasing, the proportion of the elderly in the total population is smaller compared to other developed countries, due to the high Israeli fertility rate (highest among all OECD nations, 3.08 births/woman, 2014) [3, 44].

### International comparison

Influenza vaccination rates increased since the first measurement in 2002, reaching a rate of 63.4% in 2014. Influenza vaccination rates were also measured in 33 Organization for Economic Co-operation and Development (OECD) countries. The average rate of influenza vaccination among these countries was 48.0% in 2013. In the same year, Israel’s rate was similar to the rates in Ireland (59.2%) and Canada (64.1%). The United Kingdom and United States both reached higher rates of vaccination than Israel (75.5% and 66.5%, respectively); however, many European countries such as Germany, France, and Sweden fell below Israel’s national vaccination rate at 58.6%, 51.9%, and 45.8%, respectively [45]. A recent study examining influenza vaccination rates and demographics in Austria and Croatia found that in the 2010–2011 measurement year vaccination rates were highest among the 65 years and older age group in these countries (31.1% and 45.7%, respectively) [46]. Rates of pneumococcal vaccination among Israelis aged 65–74 years increased since measurements first began in 2005, and reached a rate of 76.3% in 2014. International comparison was difficult for this indicator as quality indicator measurements of pneumococcal vaccinations on a population-wide level are limited; yet the United States measures the rate of pneumococcal vaccination in adults 65 years and older via the Healthcare Effectiveness Data and Information Set (HEDIS) survey. In 2013, 70.2% of HMO Medicare patients in the United States had previously received a pneumococcal vaccination [47]. Additionally, Australia measured the rate of adult pneumococcal vaccinations via the Adult Vaccination Survey, showing that 54.4% of Australians aged 65 years or older had previously received the pneumococcal vaccination [48]. High levels of vaccination among the elderly population promotes health maintenance by reducing influenza and pneumococcal illness, decreasing strains on the healthcare system, and providing herd immunity to those who cannot be vaccinated. While specific populations in Israel are receiving lower quality care than the majority, the overall rate in Israel indicates that more Israelis are receiving higher quality care in terms of vaccinations as compared to the international healthcare community.

The rate of body weight documentation in Israel reached a rate of 80.9% in 2015. International comparison is difficult for this measure, as most countries do not measure the rate of body weight documentation among elderly populations. However, understanding where documentation is lacking can lead to the development of protocols to increase rates in these populations, promoting better quality of care among the elderly population. Similarly, comparing the rates of underweight and significant weight-loss to rates in the elderly population in other countries is limited, as it seems that these national measures, referring to the general elderly population, are unique. However, the prevalence of underweight, as defined by BMI < 18.5 kg/m^2^, was described in large international meta-analysis including 19,538 older nursing home residents. The lowest rates were described in cohorts from Italy (4%, *n* = 181 participants), Germany (5%, *n* = 200) and Sweden (6%, *n* = 172), though an underweight rate of 10% among *n* = 1339 study participants in the USA, and 21% in China (*n* = 525), and up to 30% underweight among older nursing home residents in Japan (*n* = 8179) [32, 36].

Benzodiazepine overuse remained steady in a five-year measurement period, around 5%. Rates of long-acting benzodiazepine use decreased, achieving a rate of 2.4% in 2015. Israel measured benzodiazepine use via patient purchasing. However, many countries, including the OECD countries, measure benzodiazepine usage via rate of prescribing by the healthcare practitioner, making direct comparisons somewhat difficult. Among the countries that measure benzodiazepine use through patient medication usage, Sweden measured 11.4% of the population aged 80 years or older consumed at least one contraindicated drug during the measurement period in 2011 (benzodiazepines are included in the contraindicated drug category) [49]. In addition, an OECD 2013 study [6] measured the rates of overuse and long-acting benzodiazepine use among nine OECD countries, including Israel. The results showed the rate of benzodiazepine overuse in Israel to be 5.1%, with the highest rate of overuse in Ireland (6.3%) and the lowest rate in the Netherlands (0.7%). Among the countries studied, Israel is in the top three countries to overuse benzodiazepines, indicating poor quality of care. Furthermore, the rate of long-acting benzodiazepine use in Israel was 3.1%, with the highest rate of use in Korea (20.5%) and the lowest rate in Finland (0.5%). This comparison ranks Israel in the bottom five countries utilizing long-acting benzodiazepines, indicating fair quality of care for this indicator. While overall rates of benzodiazepine use have decreased in Israel, they are still high and of concern for the elderly population. A high rate of benzodiazepine use among the elderly indicates a health risk for this population. Identifying this risk will help to develop practitioner-focused continuing education on benzodiazepine prescribing for elderly patients.

Women received poorer quality of care in all five process indicators presented, and showed a worse picture in the two intermediate health indicators. Women were vaccinated less than males for both influenza and pneumococcal disease. These lower vaccination rates could be due to an increased negative attitude towards vaccinations and their risks among women or differences in physician recommendations between genders; however, additional evaluation is required [50]. Additionally, women overused benzodiazepines at a higher rate and used long-acting benzodiazepines more than their male counterparts; results which are consistent with prior research [51]. This is partially explained by an increase in insomnia among older women and the lack of public health concern by physicians regarding the continuous use of benzodiazepines among older adults for the treatment of insomnia [41] or the difference in how health professionals differentially diagnose and treat men versus women who present with similar symptoms [51]. Notably, the health status of the Israeli elderly was recently compared to that of the elderly in 16 European countries, showing that the Israeli elderly population is characterized with poorer health status yet a more modest gender gap, compared to their European counterparts [52].

As noted in the methods section, SEP was defined in this study according to exemption from medical co-payments. The high proportion of low SEP residents according to this definition among the elderly (39%, compared to 11% in the general population) is explained by the different, more extenuating criteria applied for elders [53].

The low SEP population overused benzodiazepines at a higher rate compared to the middle and high SEP population. This result might be influenced by the understanding that socio-economic position can frame and affect patient expectations and healthcare providers attitudes towards their patients [51]. In contrast, the low SEP population received a higher rate of pneumococcal vaccinations as compared to the high and middle SEP population. This result is possibly explained by the fact that the exempt (low SEP) elderly population has a high rate of primary care visit attendance [54], therefore providing increased opportunities for vaccination.

### Strengths and limitations

Israel’s comprehensive health insurance system allows for quality indicator development on the national level. The national health insurance provided to all eligible Israeli citizens facilitates collection of data from practically the entire Israeli population, thus providing an understanding of the quality of care for all Israelis, not simply those who have insurance. Israel’s comprehensive healthcare infrastructure includes the use of EMRs, which allows for data collection from both current and past medical histories of patients. These EMRs have been utilized for over a decade [19] creating a strong database of patient information.

However, this study does not come without limitations. First, this is an observational study suffering from the known limitations of such a design. In addition, the international comparisons were limited in their ability to make direct or exact comparisons due to the diverse nature of health care systems, as well as, different definitions of specific health concepts [20]. Notably, while this study evaluates the level of quality of care provided in the community setting to the elderly, it does not easily allow prediction of the future changes in the health status of the elderly population. Further research should be conducted to determine the extent to which changes in the quality of care affect changes in the health status. Lastly, the socio-demographic stratification was limited, as it did not stratify the data based on ethnic origin, minority classification, income level, etc.

### Scope and future development

Our study reflects the quality indicators that are currently measured in the elderly population in Israel and a question arises to what extent the existing set of quality measures represent a wider scope of quality of care for the elderly. Examining quality indicators measured in various countries, it is evident that such indicators stem from comprehensive geriatric assessment tools that intend to capture health risks and situations that impinge on quality of life. For example, an American set of measures include screening for visual problems, depression, elder abuse and urinary incontinence. In addition, some of the HMOs in Israel provide a comprehensive assessment to elderly persons considered to be at risk.

It would be, however, inappropriate to adopt many of these measures, as some of them cannot be quantified to a level that make them reliable and valid measures, and others were not shown to be clinically significant. Our program, however, is expanding as we intend to include in the near future additional measures including screening for abdominal aortic aneurysm, detection of osteoporosis and prevention of a second bone fracture, and end of life issues like the placement of advanced directives. We feel that the existing and near future measures suitably reflect the quality of care of Israel’s elderly population.

### Policy implications

Health needs of the elderly population in Israel should be met in various dimensions. The increase in the number of persons above the age of 65 beyond that of the general population makes these needs more significant and calls for thorough planning. There is a broad consensus that contrary to the expected, aging should be accompanied by good general health and control of chronic conditions, as well as being functional and enjoying good quality of life (successful aging).

The QICH program have been addressing these goals in two ways: focusing on long and short term prevention. The long-term preventive indictors encompass issues such as smoking prevalence, diabetes prevalence control and treatment of its complications and primary and secondary prevention of ischemic heart disease.

These indicators, when applied at an earlier age, may result in a healthier population in the long run. The short-term preventive indicators encompass subjects such as the use of potentially harmful medications and the detection of weight loss, which may shed light on the health status of elderly persons.

In order to improve the quality of care delivered in the community we suggest exploring additional indicators. For example, indicators that aim to eliminate hazardous life styles and ensure healthy habits between the ages of 35 to 65 should be adopted. In addition, indicators that detect frailty and counteract its bad outcomes like monitoring the risk of falls, depression and medication reviews may further protect the elderly from being dependent. Also, in light of near future global planning of moving much of the health care from hospitals to the community, indicators that monitor the quality of such transition and home care should be developed and implemented. Finally, end of life care and preparing towards end of life should also be improved by placing the quality indicators that may take the present status to a better place.

## Conclusions

Overall, healthcare quality of the Israeli elderly population has improved substantially since measurements first began; yet, it seems that females receive lower quality care than males. The overall high rates of influenza and pneumococcal vaccinations can decrease the burden of influenza and pneumococcal pneumonia on the healthcare system. In addition, high rates of body weight documentation will prompt early detection of deterioration and intervention. Higher rates of benzodiazepine usage indicate a need for continued healthcare practitioner-focused education regarding benzodiazepine prescribing in the elderly. In comparison to the international healthcare community, Israel has higher quality care for the elderly in all indicators, except for benzodiazepine overuse. Altogether, the diabetes control indicators show that the quality of care delivered to the elderly in Israel is comparable to that given to the younger population or even better.

Recognizing elderly Israeli populations who receive less than optimal care can enable further development of population-specific healthcare changes, providing quality care to specific elderly Israeli populations.

## Appendix

**Table 3:** Quality indicator performance rates stratified by gender and socioeconomic position, 2015

Influenza vaccination										
		Male			Female			Total		
SEP	Age	Numerator	Denominator	Rate	Numerator	Denominator	Rate	Numerator	Denominator	Rate
Low SEP	65–74	45,214	70,197	64.41%	54,684	94,383	57.94%	99,898	164,580	60.70%
	75–84	35,469	50,525	70.20%	47,185	75,635	62.39%	82,654	126,160	65.52%
	85 or above	12,234	16,742	73.07%	21,008	32,913	63.83%	33,242	49,655	66.95%
	total	92,917	137,464	67.59%	122,877	202,931	60.55%	215,794	340,395	63.40%
Middle & high SEP	65–74	93,556	156,522	59.77%	97,471	167,895	58.05%	191,027	324,417	58.88%
	75–84	50,355	69,352	72.61%	59,586	87,697	67.95%	109,941	157,049	70.00%
	85 or above	16,090	22,154	72.63%	23,503	35,656	65.92%	39,593	57,810	68.49%
	total	160,001	248,028	64.51%	180,560	291,248	62.00%	340,561	539,276	63.15%
Total		252,918	385,492	65.61%	303,437	494,179	61.40%	556,355	879,671	63.25%
Pneumococcal vaccination										
		Male			Female			Total		
SEP	Age	Numerator	Denominator	Rate	Numerator	Denominator	Rate	Numerator	Denominator	Rate
Low SEP	65–74	49,631	59,066	84.03%	60,870	77,072	78.98%	110,501	136,138	81.17%
	75–84	NA	NA	NA	NA	NA	NA	NA	NA	NA
	85 or above	NA	NA	NA	NA	NA	NA	NA	NA	NA
	total	49,631	59,066	84.03%	60,870	77,072	78.98%	110,501	136,138	81.17%
Middle & high SEP	65–74	164,901	208,638	79.04%	181,131	240,636	75.27%	346,032	449,274	77.02%
	75–84	NA	NA	NA	NA	NA	NA	NA	NA	NA
	85 or above	NA	NA	NA	NA	NA	NA	NA	NA	NA
	total	164,901	208,638	79.04%	181,131	240,636	75.27%	346,032	449,274	77.02%
Total		214,532	267,704	80.14%	242,001	317,708	76.17%	456,533	585,412	77.98%
Benzodiazepine overuse										
		Male			Female			Total		
SEP	Age	Numerator	Denominator	Rate	Numerator	Denominator	Rate	Numerator	Denominator	Rate
Low SEP	65–74	2698	70,197	3.84%	4350	94,383	4.61%	7048	164,580	4.28%
	75–84	3435	50,525	6.80%	6775	75,635	8.96%	10,210	126,160	8.09%
	85 or above	2021	16,742	12.07%	4562	32,913	13.86%	6583	49,655	13.26%
	total	8154	137,464	5.93%	15,687	202,931	7.73%	23,841	340,395	7.00%
Middle & high SEP	65–74	2639	156,522	1.69%	4880	167,895	2.91%	7519	324,417	2.32%
	75–84	2888	69,352	4.16%	5848	87,697	6.67%	8736	157,049	5.56%
	85 or above	1721	22,154	7.77%	4041	35,656	11.33%	5762	57,810	9.97%
	total	7248	248,028	2.92%	14,769	291,248	5.07%	22,017	539,276	4.08%
Total		15,402	385,492	4.00%	30,456	494,179	6.16%	45,858	879,671	5.21%
Long-term benzodiazepine										
		Male			Female			Total		
SEP	Age	Numerator	Denominator	Rate	Numerator	Denominator	Rate	Numerator	Denominator	Rate
Low SEP	65–74	1675	70,197	2.39%	2567	94,383	2.72%	4242	164,580	2.58%
	75–84	1180	50,525	2.34%	2297	75,635	3.04%	3477	126,160	2.76%
	85 or above	424	16,742	2.53%	896	32,913	2.72%	1320	49,655	2.66%
	total	3279	137,464	2.39%	5760	202,931	2.84%	9039	340,395	2.66%
Middle & high SEP	65–74	2544	156,522	1.63%	4163	167,895	2.48%	6707	324,417	2.07%
	75–84	1478	69,352	2.13%	2713	87,697	3.09%	4191	157,049	2.67%
	85 or above	500	22,154	2.26%	1032	35,656	2.89%	1532	57,810	2.65%
	total	4522	248,028	1.82%	7908	291,248	2.72%	12,430	539,276	2.30%
Total		7801	385,492	2.02%	13,668	494,179	2.77%	21,469	879,671	2.44%
BMI documentation										
		Male			Female			Total		
SEP	Age	Numerator	Denominator	Rate	Numerator	Denominator	Rate	Numerator	Denominator	Rate
Low SEP	65–74	53,898	64,758	83.23%	70,201	85,632	81.98%	124,099	150,390	82.52%
	75–84	40,220	46,809	85.92%	57,509	69,604	82.62%	97,729	116,413	83.95%
	85 or above	NA	NA	NA	NA	NA	NA	NA	NA	NA
	total	94,118	111,567	84.36%	127,710	155,236	82.27%	221,828	266,803	83.14%
Middle & high SEP	65–74	125,290	160,318	78.15%	137,598	174,765	78.73%	262,888	335,083	78.45%
	75–84	61,741	74,009	83.42%	77,274	94,670	81.62%	139,015	168,679	82.41%
	85 or above	NA	NA	NA	NA	NA	NA	NA	NA	NA
	total	187,031	234,327	79.82%	214,872	269,435	79.75%	401,903	503,762	79.78%
Total		281,149	345,894	81.28%	342,582	424,671	80.67%	623,731	770,565	80.94%
Underweight										
		Male			Female			Total		
SEP	Age	Numerator	Denominator	Rate	Numerator	Denominator	Rate	Numerator	Denominator	Rate
Low SEP	65–74	6122	53,898	11.36%	6816	70,201	9.71%	12,938	124,099	10.43%
	75–84	5127	40,220	12.75%	6426	57,509	11.17%	11,553	97,729	11.82%
	85 or above	2511	13,178	19.05%	4453	22,450	19.84%	6964	35,628	19.55%
	total	13,760	107,296	12.82%	17,695	150,160	11.78%	31,455	257,456	12.22%
Middle & high SEP	65–74	11,220	125,261	8.96%	19,388	137,576	14.09%	30,608	262,837	11.65%
	75–84	7874	62,120	12.68%	11,326	77,765	14.56%	19,200	139,885	13.73%
	85 or above	3683	17,688	20.82%	5686	25,961	21.90%	9369	43,649	21.46%
	total	22,777	205,069	11.11%	36,400	241,302	15.08%	59,177	446,371	13.26%
Total		36,537	312,365	11.70%	54,095	391,462	13.82%	90,632	703,827	12.88%
Weight loss										
		Male			Female			Total		
SEP	Age	Numerator	Denominator	Rate	Numerator	Denominator	Rate	Numerator	Denominator	Rate
Low SEP	65–74	2694	44,749	6.02%	3637	57,831	6.29%	6331	102,580	6.17%
	75–84	2417	36,760	6.58%	4038	52,070	7.75%	6455	88,830	7.27%
	85 or above	923	11,934	7.73%	1904	19,601	9.71%	2827	31,535	8.96%
	total	6034	93,443	6.46%	9579	129,502	7.40%	15,613	222,945	7.00%
Middle & high SEP	65–74	4128	103,165	4.00%	5706	114,904	4.97%	9834	218,069	4.51%
	75–84	3398	58,820	5.78%	5062	73,364	6.90%	8460	132,184	6.40%
	85 or above	1408	16,582	8.49%	2358	23,628	9.98%	3766	40,210	9.37%
	total	8934	178,567	5.00%	13,126	211,896	6.19%	22,060	390,463	5.65%
Total		14,968	272,010	5.50%	22,705	341,398	6.65%	37,673	613,408	6.14%

## Abbreviations

CBS: Central Bureau of Statistics
DDD: Defined Daily Dose
EMR: Electronic Medical Record
GSA: Geographical statistical area
HEDIS: Health Effectiveness Data and Information Set
HMO: Health Maintenance Organization
ILI: Influenza-like Illness
IPD: Invasive Pneumococcal Disease
NHI: National Health Insurance
OECD: Organisation for Economic Co-operation and Development
QICH: Israel National Program for Quality Indicators in Community Healthcare
SEP: Socio-Economic Position
WHO: World Health Organization
