# Correction to: The quality of primary care provided to the elderly in Israel

**DOI:** 10.1186/s13584-021-00457-x

**Published:** 2021-03-10

**Authors:** Rachel Podell, Vered Kaufman-Shriqui, Yael Wolff Sagy, Orly Manor, Arie Ben-Yehuda

**Affiliations:** 1 Program team of the National Program for Quality Indicators in Community Healthcare in Israel, Hebrew University, POB 12272, 92210 Jerusalem, Israel; 2 Braun School of Public Health, Hebrew University, POB 12272, 92210 Jerusalem, Israel; 3 Department of Nutritional Sciences, School of Health Sciences, Ariel University, Ariel, Israel; 4 Program directorate of the National Program for Quality Indicators in Community Healthcare in Israel, Hebrew University, POB 12272, 92210 Jerusalem, Israel; 5 Braun School of Public Health, Hebrew University, POB 12272, 92210 Jerusalem, Israel; 6 Program directorate of the National Program for Quality Indicators in Community Healthcare in Israel, Hadassah Medical Center, POB 12000, 92210 Jerusalem, Israel

**Correction to: Isr J Health Policy Res 7, 21 (2018)**

**https://doi.org/10.1186/s13584-018-0214-3**

Following publication of the original article [1], the authors identified an error in the author name of Vered Kaufman-Shriqui. “Kaufman” was incorrectly shown as a given name and the hyphen was missing.

The author group has been updated above and the original article [1] has been corrected.
